# Impact of lung function decline on time to hospitalisation events in systemic sclerosis-associated interstitial lung disease (SSc-ILD): a joint model analysis

**DOI:** 10.1186/s13075-021-02710-9

**Published:** 2022-01-10

**Authors:** Michael Kreuter, Francesco Del Galdo, Corinna Miede, Dinesh Khanna, Wim A. Wuyts, Laura K. Hummers, Margarida Alves, Nils Schoof, Christian Stock, Yannick Allanore

**Affiliations:** 1 Center for Interstitial and Rare Lung Diseases, Pneumology and Respiratory Care Medicine, Thoraxklinik, University of Heidelberg, Röntgenstrasse 1, 69121 Heidelberg, Germany; 2 German Center for Lung Research (DZL), Heidelberg, Germany; 3 Scleroderma Programme NIHR BRC and Institute of Rheumatic and Musculoskeletal Medicine, University of Leeds, Leeds, UK; 4 mainanalytics GmbH, Sulzbach/Taunus, Germany; 5 Division of Rheumatology/Department of Internal Medicine, Scleroderma Program, University of Michigan, Ann Arbor, MI USA; 6 Interstitial Lung Diseases Unit, University Hospitals Leuven, Leuven, Belgium; 7 Division of Rheumatology, Johns Hopkins University School of Medicine, Baltimore, MD USA; 8 Boehringer Ingelheim International GmbH, Ingelheim am Rhein, Germany; 9 Boehringer Ingelheim Pharma GmbH & Co. KG, Ingelheim am Rhein, Germany; 10 Department of Rheumatology A, Descartes University, APHP, Cochin Hospital, Paris, France

**Keywords:** Joint model, SENSCIS, Systemic sclerosis-associated interstitial lung disease, Surrogate endpoint, Hospitalisation, Forced vital capacity

## Abstract

**Background:**

Interstitial lung disease (ILD) is a common organ manifestation in systemic sclerosis (SSc) and is the leading cause of death in patients with SSc. A decline in forced vital capacity (FVC) is an indicator of ILD progression and is associated with mortality in patients with SSc-associated ILD (SSc-ILD). However, the relationship between FVC decline and hospitalisation events in patients with SSc-ILD is largely unknown. The objective of this post hoc analysis was to investigate the relationship between FVC decline and clinically important hospitalisation endpoints.

**Methods:**

We used data from SENSCIS®, a phase III trial investigating the efficacy and safety of nintedanib in patients with SSc-ILD. Joint models for longitudinal and time-to-event data were used to assess the association between rate of decline in FVC% predicted and hospitalisation-related endpoints (including time to first all-cause hospitalisation or death; time to first SSc-related hospitalisation or death; and time to first admission to an emergency room [ER] or admission to hospital followed by admission to intensive care unit [ICU] or death) during the treatment period, over 52 weeks in patients with SSc-ILD.

**Results:**

There was a statistically significant association between FVC decline and the risk of all-cause (*n* = 78) and SSc-related (*n* = 42) hospitalisations or death (both *P* < 0.0001). A decrease of 3% in FVC corresponded to a 1.43-fold increase in risk of all-cause hospitalisation or death (95% confidence interval [CI] 1.24, 1.65) and a 1.48-fold increase in risk of SSc-related hospitalisation or death (95% CI 1.23, 1.77). No statistically significant association was observed between FVC decline and admission to ER or to hospital followed by admission to ICU or death (*n* = 75; *P* = 0.15). The estimated slope difference for nintedanib versus placebo in the longitudinal sub-model was consistent with the primary analysis in SENSCIS®.

**Conclusions:**

The association of lung function decline with an increased risk of hospitalisation suggests that slowing FVC decline in patients with SSc-ILD may prevent hospitalisations. Our findings also provide evidence that FVC decline may serve as a surrogate endpoint for clinically relevant hospitalisation-associated endpoints.

**Trial registration:**

ClinicalTrials.govNCT02597933. Registered on 8 October 2015.

**Supplementary Information:**

The online version contains supplementary material available at 10.1186/s13075-021-02710-9.

## Background

Systemic sclerosis (SSc) is a chronic autoimmune disease characterised by extensive fibrosis of the skin, internal organs and vasculopathy [1]. Interstitial lung disease (ILD) is a common manifestation of SSc, in which parenchymal involvement can lead to pulmonary fibrosis and declining pulmonary function [2, 3]. SSc-associated ILD (SSc-ILD) is associated with reduced survival and is the leading cause of death in patients with SSc [4].

Forced vital capacity (FVC) is currently supported as an endpoint for randomised controlled trials and is the primary outcome measure in many of them [5, 6]. An FVC absolute decline of at least 10% is an established measure of progression in more progressive ILDs like idiopathic pulmonary fibrosis (IPF) [7], but lower levels of progression in SSc-ILD may better reflect the disease course. For instance, an FVC absolute decline of 5–9% with a diffusing capacity of the lung for carbon monoxide (DL_CO_) absolute decline of ≥ 15% has been considered optimal for trial purposes [8], and an FVC decline or improvement of approximately 3% was considered a minimal clinically important difference based on data from Scleroderma Lung Study I and II [6].

Although FVC is important, patient-reported outcome (PRO) measures have recently received growing recognition as they can provide valuable insights about the impact of a disease on patients and drive improvements in care [9]. Studies have shown that cough, dyspnoea, changes in physical function and fatigue are the lung-related symptoms of most concern to patients with connective tissue disease (CTD)-associated ILD, including SSc [10, 11]. Thus, PRO measures are usually assessed in randomised clinical trials in SSc-ILD as secondary endpoints [12].

SENSCIS® was a large, phase III, randomised, placebo-controlled, parallel trial of nintedanib, a multi-target tyrosine kinase inhibitor with anti-inflammatory and antifibrotic properties, in patients with SSc-ILD [13]. SENSCIS® demonstrated a statistically significant 44% reduction in ILD progression in patients with SSc-ILD receiving nintedanib compared with those who received placebo. For PRO measures, very small changes were observed over 52 weeks, with no differences between nintedanib and placebo. Post hoc analysis showed that patients with more impaired lung function (FVC < 70% predicted at baseline) had worse health-related quality of life (HRQoL; as indicated by higher St. George’s Respiratory Questionnaire values) and that a moderate or large decline in lung function was associated with a more pronounced decline in HRQoL [14]. Nevertheless, defining relationships between pulmonary function tests and PRO measures remains a significant challenge in ILD.

Hospitalisation is recognised as a serious event by both patients and physicians, and has clear health-economic implications. The frequency of hospitalisations and emergency room (ER) visits was considered important to a CTD-ILD patient focus group [10]. Furthermore, longitudinal data from the German INSIGHTS-IPF registry suggested an association between hospitalisation and poorer HRQoL in patients with IPF [15]. Existing data have demonstrated that patients with SSc-ILD have a higher economic burden compared with patients with other organ involvement associated with SSc, which has been largely attributed to inpatient hospitalisations [3]. In addition, several US-based studies have agreed that SSc-ILD is associated with greater healthcare costs compared with SSc alone [16–18]. For patients with incident SSc-ILD, healthcare costs in the USA are nearly twice as high over 5 years compared with patients with incident SSc [16].

In cases where clinically relevant endpoints are difficult to measure or particularly rare in clinical trials, surrogate endpoints such as biomarkers may be a suitable substitute if the two endpoints are known to be correlated. This is also an option for hospitalisation and mortality outcomes in slow, progressive diseases such as SSc-ILD, where large sample sizes and long follow-up times would be required due to low event incidence [19]. A biomarker of SSc-ILD progression such as FVC decline may be able to serve as a surrogate for hospitalisation endpoints if a relationship could be established.

Joint modelling allows for the combined analysis of longitudinal and time-to-event endpoints in a single model and the assessment of the validity of the potential surrogate endpoint [20, 21]. If an association is identified and a treatment effect on the surrogate endpoint is also established, this provides evidence of an indirect treatment effect on the clinically important endpoint [22].

The objective of this post hoc analysis was to investigate the association between longitudinal FVC decline and time to hospitalisation endpoints in SSc-ILD using data from the SENSCIS® trial, and thus to gain insights into the validity of FVC as a surrogate marker for hospitalisation in SSc-ILD.

## Methods

### Data source

The design and eligibility criteria for SENSCIS® have been reported previously [13]. Briefly, adult patients with SSc [23] were enrolled if they had disease onset (first non-Raynaud symptom of SSc) within the previous 7 years, evidence of lung fibrosis in ≥ 10% of the lung using high-resolution computed tomography, an FVC ≥ 40% of the predicted value, and haemoglobin-corrected DL_CO_ 30–89% of the predicted value. Evidence of previous ILD progression was not required for entry into SENSCIS®. Eligible patients were randomised in a double-blind fashion to receive either nintedanib (*n* = 288) or placebo (*n* = 288) for up to 100 weeks or until the last randomised patient reached 52 weeks of treatment, with the primary efficacy evaluation (annual rate of decline in FVC [mL/year]) conducted at 52 weeks. Concomitant treatment with prednisone ≤ 10 mg/day (or equivalent) and/or stable therapy with mycophenolate or methotrexate for ≥ 6 months prior to randomisation was allowed. Additional therapy was allowed during the treatment period if clinically significant deterioration occurred. Patients were stratified according to the presence of anti-topoisomerase I antibody (ATA), which is associated with declining FVC in patients with early SSc [13, 24].

### Included time-to-event and longitudinal endpoints

The two outcome variables considered in this study and analysed together in a joint model were defined as follows. Various time to hospitalisation endpoints in the context of the SENSCIS® trial period (over 52 weeks and the whole trial) were considered for inclusion in a joint model. These were (1) time to first all-cause hospitalisation or death, (2) time to first SSc-related hospitalisation or death, (3) time to first admission to the ER or admission to hospital followed by admission to an intensive care unit (ICU) or death, (4) time to first admission to hospital followed by admission to ICU or death, and (5) time to first admission to hospital followed by use of mechanical ventilation or death. Hospitalisation-associated events, including those associated with SSc, were reported and verified by clinicians. Only hospitalisations that occurred during the treatment period or 28 days thereafter were considered.

The longitudinal response variable was the rate of decline in FVC% predicted over 52 weeks and the whole trial. Of note, the rate used here is a model-based estimate that does not require a full observation time of 52 weeks. Only values obtained from on-treatment assessments were considered for inclusion. In a secondary analysis, we further considered the FVC% predicted at a given time (i.e. irrespective of the slope) as the longitudinal response. FVC% predicted was preferred over FVC measured in mL because it already provides an adjustment for prognostic variables, which simplifies the longitudinal modelling.

### Statistical analysis

The total sample and subsamples of patients with and without hospitalisation events were characterised using standard descriptive statistics. Joint modelling for longitudinal and time-to-event data was used to assess the association between rate of decline in FVC% predicted and hospitalisation-related endpoints [19, 20]. The approach was implemented using the SAS® macro %JM [25], and validated using the R package JM, version 1.4-8 [26].

#### Longitudinal sub-model

We used a normal mixed effects model of FVC% predicted with ATA status and FVC% predicted at baseline as predictor variables. Separate mean slopes for patients on nintedanib treatment and controls were assumed, and trajectories were modelled by a linear trend with an unstructured variance–covariance matrix assumed, given the known effect of nintedanib on FVC. All FVC values in the SENSCIS® trial were used: visits at 2, 4, 6, 12, 24, 36 and 52 weeks (visits 3 to 9) for the 52-week time period and visits at 68, 84 and 100 weeks (visits 10 to 12) for whole trial period. Only on-treatment FVC measurements before a hospitalisation event were considered.

#### Time to event sub-model

For the time to event sub-model, a piecewise exponential model with five internal knots was used to model the baseline hazard. This is a standard approach used in joint modelling of longitudinal and time-to-event data where the baseline hazard is usually modelled explicitly. It assumes that hazards are constant over the particular time intervals and avoids misspecification of time-to-event distribution. FVC% predicted (i.e. the longitudinal response) was used as the endogenous time-dependent covariate, and the time to event sub-model was additionally stratified by ATA status. Of interest was the time to the first event in any of the components of the composite. Patients who experienced an event were censored afterwards and not at risk of further events. This excludes the possibility that events have an impact on the underlying FVC trajectories and thereby potential reverse causality bias.

### Association structure

The shared parameter in each of the joint models was FVC% predicted, which was considered as either the “estimated slope” (assuming the rate of FVC% predicted affected the risk of an event occurring; primary analysis) or the “estimated current value” (assuming the risk of a hospitalisation event at a given time depended on the estimated value of FVC% predicted at that time; secondary analysis). Importantly, the estimated slope represents the annual rate of decline in FVC% predicted. It is derived based on the whole estimated trajectory of a patient and allowed to vary over time, and thus is always an estimate referring to a specific point in time. In compliance with recommended reporting, all model coefficients and their precisions are available in Supplementary Methods S1–6 [27].

For each hospitalisation endpoint observed during the SENSCIS® trial with sufficient numbers of events across two time periods (52 weeks and whole trial), joint models were fitted. We present the risk of first hospitalisation events in terms of 1-, 3- and 5-unit FVC decline, as well as graphically from > 0 to 10-unit FVC decline. A “unit” here represents 1 percentage point. Results with a *P *value ≤ 0.05 were considered statistically significant.

## Results

### Participant characteristics

A total of 576 participants were enrolled in the SENSCIS® trial and received at least one dose of nintedanib or placebo. FVC and hospitalisation data were available for 574 patients and were therefore included in this analysis. The baseline characteristics of all patients included in the joint models and those with a hospitalisation event or death during the trial period are described in Table 1. Most patients were female (75.1%). Approximately 50% of patients had diffuse cutaneous SSc and 60.8% of the total population were ATA positive. The mean FVC% predicted (± standard deviation [SD]) at baseline was 72.5 ± 16.7 for the total population, 72.0 ± 16.4 and 70.5 ± 18.7 for patients with all-cause and SSc-related hospitalisation events or death and 70.9 ± 16.7 for patients with ER or hospital admission followed by admission to ICU or death. The annual rate of decline in FVC% predicted for patients enrolled in SENSCIS® was − 1.4 ± 0.4% and − 2.6 ± 0.4% for nintedanib and placebo, respectively. Baseline comorbidities and SSc-related organ manifestations, as well as discontinuation data at 52 weeks, are available in Supplementary Results Tables S7–9.

**Table 1:** Summary of baseline demographics of patients in the SENSCIS® trial

	Patients included in joint models (***N*** = 574)^**a**^	Patients with all-cause hospitalisation events or death (***n*** = 78)	Patients with SSc-related hospitalisation events or death (***n*** = 42)	Patients with admission to ER or hospital followed by admission to ICU or death (***n*** = 75)
Sex, *n* (%)				
Female	431 (75.1)	50 (64.1)	28 (66.7)	51 (68.0)
Age, years	53.9 ± 12.2	56.1 ± 12.0	56.1 ± 12.0	54.4 ± 11.6
Diffuse cutaneous SSc, *n* (%)	298 (51.9)	39 (50.0)	21 (50.0)	38 (50.7)
Time since the onset of the first non-Raynaud’s symptom, years	3.5 ± 1.7	3.5 ± 1.9	3.2 ± 2.0	3.6 ± 1.7
Extent of fibrosis of the lungs on HRCT, %	36.0 ± 21.2	35.9 ± 20.2	38.5 ± 21.2	36.3 ± 21.9
FVC, mL	2501.2 ± 778.2	2532.0 ± 787.2	2498.2 ± 817.2	2455.7 ± 693.5
FVC, % predicted	72.5 ± 16.7	72.0 ± 16.4	70.5 ± 18.7	70.9 ± 16.7
DL_CO_, % predicted^b^	53.1 ± 15.1	48.4 ± 13.2	47.7 ± 12.6	49.2 ± 13.5
ATA positive, *n* (%)^c^	349 (60.8)	42 (53.8)	24 (57.1)	43 (57.3)
mRSS^d^	11.1 ± 9.0	12.3 ± 11.1	13.2 ± 11.6	11.6 ± 9.0
Total score on the SGRQ^e^	39.9 ± 20.4	44.3 ± 18.8	41.3 ± 19.9	47.9 ± 17.9
High-sensitivity C-reactive protein, mg/L^f^	6.2 ± 15.2	10.6 ± 30.4	14.5 ± 40.5	10.6 ± 31.4

Data are mean ± SD unless otherwise stated

^a^Data on some variables were not available for all patients

^b^The DL_CO_ value was corrected for the haemoglobin level. DL_CO_ values were available for 567 patients in total, 77, 41 and 74 patients with all-cause hospitalisation events or death, SSc-related hospitalisation events or death, and admission to ER or hospital followed by admission to ICU or death, respectively

^c^Historical information on ATA status was used, or, if this information was not available to the trial sites, it was provided by a central laboratory

^d^Scores were available for 572 patients in total, 77 and 74 patients with all-cause hospitalisation events or death, and admission to ER or hospital followed by admission to ICU or death, respectively

^e^Total scores on the SGRQ range from 0 to 100, with higher scores indicating worse health-related quality of life. Scores were available for 563 patients in total, 75 and 73 in patients with all-cause hospitalisation events or death, and admission to ER or hospital followed by admission to ICU or death, respectively

^f^High-sensitivity C-reactive protein values were available for 529 patients in total, 71, 39 and 66 patients with all-cause hospitalisation events or death, SSc-related hospitalisation events or death, and admission to ER or hospital followed by admission to ICU or death, respectively

*ATA,* anti-topoisomerase I antibody, *DL*_*CO,*_ diffusing capacity of the lungs for carbon monoxide, *ER,* emergency room, *FVC,* forced vital capacity, *HRCT,* high-resolution computed tomography, *ICU,* intensive care unit, *mRSS,* modified Rodnan Skin Score, *SD,* standard deviation, *SGRQ,* St. George’s Respiratory Questionnaire, *SSc,* systemic sclerosis

### Joint model analysis

Six joint models using the estimated slope of FVC decline as the shared parameter were fitted. Low numbers of reported events precluded stable model fits for the endpoints time to first admission to hospital followed by admission to ICU or death (*n* = 11) and time to first admission to hospital followed by use of mechanical ventilation or death (*n* = 7).

At 52 weeks, 78 (13.7%) patients either had an all-cause hospitalisation event or died. Of these, 42 experienced a hospitalisation event or death related to SSc, accounting for 7.4% of patients included in the respective joint model. Seventy-five patients were admitted to ER or hospital (including all-cause hospitalisation patients) followed by ICU or death, accounting for 13.1% of patients included in the respective joint model (Table 2).

**Table 2:** Association between slope of FVC% predicted and risk of first hospitalisation endpoints over 52 weeks

	Time to first all-cause hospitalisation or death (***n*** = 568)	Time to first SSc-related hospitalisation or death (***n*** = 570)	Time to first admission to ER or admission to hospital followed by admission to ICU or death (***n*** = 572)
**Longitudinal sub-model**^**a**^			
Estimated slope difference nintedanib vs. placebo (95% CI)	1.16 (0.00, 2.32)	1.44 (0.33, 2.55)	1.33 (0.18, 2.48)
*P* value	0.0497	0.01	0.02
**Time to event sub-model**^**b**^			
Number of patients with event, *n* (%)	78 (13.7)	42 (7.4)	75 (13.1)
**Difference in estimated slope of FVC% predicted, HR (95% CI)**			
1-unit decrease	1.13 (1.07, 1.18)	1.14 (1.07, 1.21)	1.05 (0.98, 1.12)
3-unit decrease	1.43 (1.24, 1.65)	1.48 (1.23, 1.77)	1.15 (0.95, 1.41)
5-unit decrease	1.81 (1.42, 2.30)	1.91 (1.41, 2.60)	1.27 (0.91, 1.76)
*P* value	< 0.0001	< 0.0001	0.15

Data collected during the treatment period

^a^Random effects normal linear model of FVC% predicted with predictor variables ATA status and FVC% predicted at baseline, a separate slope for patients on treatment, trajectories modelled by a linear trend, and an unstructured variance–covariance matrix

^b^Piecewise exponential baseline hazard, stratified by ATA status, and endogenous time-dependent covariate FVC% predicted as estimated slope of the longitudinal response

*ATA*, anti-topoisomerase antibody; *CI*, confidence interval; *ER*, emergency room; *FVC*, forced vital capacity; *HR*, hazard ratio; *ICU*, intensive care unit; *SSc*, systemic sclerosis

**Fig. 1:**
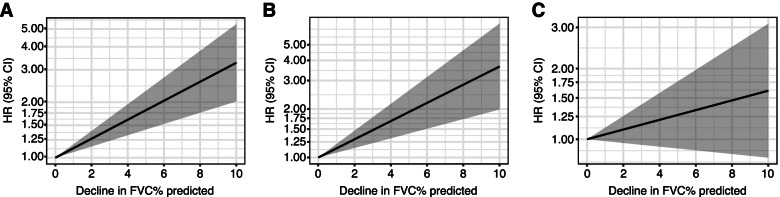
Change in risk of first hospitalisation endpoints by decline in FVC% predicted **a** all-cause hospitalisation or death, **b** SSc-related hospitalisation or death and **c** ER or hospital admission followed by ICU or death. Data collected during the treatment period over 52 weeks. *CI*, confidence interval; *ER*, emergency room; *FVC*, forced vital capacity; *HR*, hazard ratio; *ICU*, intensive care unit; *SSc*, systemic sclerosis

We observed a statistically significant relationship between FVC decline and time to first all-cause hospitalisation or death during 52 weeks of the SENSCIS® trial, with a 3-unit decline in FVC% predicted corresponding to a 1.43-fold increase in the risk of an event (hazard ratio [HR] 1.43; 95% confidence interval [CI] 1.24, 1.65; *P* < 0.0001; Table 2). These values were consistent with those calculated over the whole trial (3-unit FVC% predicted decline: 1.47-fold increased risk; 95% CI 1.25, 1.74; *P* < 0.0001; Table 3). FVC decline was also significantly associated with time to first SSc-related hospitalisation or death during 52 weeks of the SENSCIS® trial, with a 3-unit decline in FVC% predicted corresponding to a 1.48-fold increase in the risk of an event (HR 1.48; 95% CI 1.23, 1.77; *P* < 0.0001). Corresponding data for 1- and 5-unit FVC% predicted declines are shown in Table 2. These findings were consistent with those calculated over the whole trial (3-unit FVC% predicted decline: 1.60-fold increased risk; 95% CI 1.29, 1.98; *P* < 0.0001; Table 3). There was no association found between FVC decline and the time to first admission to ER or admission to hospital followed by admission to ICU or death at either 52 weeks or whole trial (*P* = 0.1549 and *P* = 0.3376, respectively). HRs for varying values of FVC decline are visualised in Fig. 1. The estimated slope difference for nintedanib versus placebo in the longitudinal FVC% predicted sub-model was statistically significant at both time points, indicating that the rate of FVC decline was slower with nintedanib (Tables 2 and 3).

**Table 3:** Association between slope of FVC% predicted and risk of first hospitalisation endpoints over the whole trial

	Time to first hospitalisation or death (***n*** = 568)	Time to first SSc-related hospitalisation or death (***n*** = 570)	Time to first admission to ER or admission to hospital followed by admission to ICU or death (***n*** = 572)
**Longitudinal sub-model**^**a**^			
Estimated slope difference nintedanib vs. placebo (95% CI)	1.07 (0.10, 2.05)	1.19 (0.27, 2.12)	1.18 (0.23, 2.13)
*P* value	0.03	0.01	0.02
**Time to event sub-model**^**b**^			
Number of patients with event, *n* (%)	103 (18.1)	56 (9.8)	90 (15.7)
**Difference in estimated slope of FVC% predicted, HR (95% CI)**			
1-unit decrease	1.14 (1.08, 1.20)	1.17 (1.09, 1.26)	1.04 (0.96, 1.13)
3-unit decrease	1.47 (1.25, 1.74)	1.60 (1.29, 1.98)	1.12 (0.89, 1.43)
5-unit decrease	1.91 (1.44, 2.51)	2.18 (1.52, 3.13)	1.21 (0.82, 1.81)
*P* value	< 0.0001	< 0.0001	0.34

Data collected during the treatment period

^a^Random effects normal linear model of FVC% predicted with predictor variables ATA status and FVC% predicted at baseline, a separate slope for patients on treatment, trajectories modelled by a linear trend, and an unstructured variance−covariance matrix

^b^Piecewise exponential baseline hazard, stratified by ATA status, and endogenous time-dependent covariate FVC% predicted as estimated slope of the longitudinal response

*ATA*, anti-topoisomerase antibody; *CI*, confidence interval; *ER*, emergency room; *FVC*, forced vital capacity; *HR*, hazard ratio; *ICU*, intensive care unit; *SSc*, systemic sclerosis

In additional joint models using the estimated current value of FVC% predicted as the shared parameter (which ignore the individual dynamics), FVC was not statistically significantly associated with the risk of any hospitalisation events or death over 52 weeks (all-cause: *P* = 0.29; SSc-related: *P* = 0.13; admission to ER or hospital followed by ICU: *P* = 0.22; Supplementary Results S10).

## Discussion

To the best of our knowledge, this is the first study to assess the impact of FVC changes on the risk of hospitalisation in SSc-ILD. Our joint model approach using data from the SENSCIS® trial showed that a decline in FVC% predicted corresponded to a statistically significant increase in the risk of all-cause and SSc-related hospitalisation or death during the treatment period. This is a new finding in SSc and is consistent with data in IPF [28]; it is also clinically important since hospitalisation rate is one of the most important outcomes for patients. We also observed a statistically significant treatment effect of nintedanib on FVC decline, mirroring the results from the SENSCIS® trial in which nintedanib reduced the rate of FVC decline compared with placebo [13]. Our findings suggest that even small reductions in the decline of FVC% predicted (1–3 units) in response to a treatment intervention can result in relevant reductions in the risk of hospitalisation.

There was no association between the rate of FVC decline and the risk of admission to ER or hospital followed by admission to ICU or death. Although ILD is part of a series of systemic complications in SSc that can lead to hospitalisations, the proportion of ILD-related hospital or ER admissions was not recorded in SENSCIS®, limiting further analysis of this finding. In addition, patients may have required hospitalisation for procedures and special medication administration, but this was also not recorded.

The baseline characteristics of all patients with hospitalisation events were similar to the overall population. Baseline FVC% predicted and DL_CO_ values were numerically slightly lower in patients with hospitalisation events compared with the overall population. Over 20% of hospitalised patients had infections and infestations at baseline, which may be related to concomitant immunosuppression or more severe disease. Furthermore, the trial discontinuation rate was higher in patients who had an all-cause or SSc-related hospitalisation or death than in the overall population. Patients with all-cause or SSc-related hospitalisation events had numerically higher C-reactive protein levels and a higher proportion had elevated creatine kinase levels compared with the overall population. It is possible that patients who had an SSc-related hospitalisation or death may have had more complications related to more active inflammatory disease.

Our analysis used two different parameterisations of the association structure in the joint model. Importantly, we observed strong evidence in the primary analysis that the slope of the FVC decline is predictive of hospitalisation, whereas a secondary analysis exhibited no (or very little) evidence of the current FVC value as a predictor of hospitalisation. As an implication for clinical practice, this suggests that the dynamics of the decline in FVC play a larger role than singular FVC values in the assessment of hospitalisation risk. It also underlines the importance of monitoring changes in FVC over time in clinical practice to assess the hospitalisation risk of individual patients.

Given the potential relationship between the change in FVC and the risk of hospitalisation, we evaluated the validity of FVC as a surrogate endpoint for the clinically important hospitalisation-associated endpoints according to the three levels of surrogacy defined by Taylor and Elston [22]. Since we have observed a statistically significant association between the surrogate outcome of FVC decline and time to hospitalisation events (which is a patient-related outcome), our findings meet the second, intermediate level of the endpoint surrogacy criteria. With an established link between these outcomes, further investigations are required to evaluate whether the slowing effect of nintedanib on FVC decline directly corresponds to a reduction in the risk of a hospitalisation event.

Our study has the following limitations. First, the analysis was limited to the data collected in the SENSCIS® trial, where data on the reasons for hospital admissions were not specific and were limited to whether they were SSc-related or not. A basic linkage with adverse events leading to hospitalisation recorded in temporal proximity to hospitalisations suggests that the following system organ classes were most frequently associated with hospitalisations: “respiratory, thoracic and mediastinal disorders” and “infections and infestations” (each approximately 20% of admissions) and “gastrointestinal disorders” (approximately 10% of admissions). However, these observations need to be treated with caution due to imperfect linkage and missing data. A further limitation is that data collection was restricted to the duration of the clinical trial. Second, the SSc-ILD population had relatively preserved lung function and many were receiving immunosuppressant therapy, which may have slowed down ILD progression but also may have increased the risk for infection-related hospitalisations. Third, as the study was performed in an SSc-ILD population, generalisability to an overall SSc population may be limited. Finally, since these results were obtained from a controlled clinical trial environment, replication in clinical practice would be desirable as the association between FVC decline reduction and risk of hospitalisation reduction is of great clinical importance.

## Conclusions

In conclusion, our joint modelling approach indicated that a decline in FVC has a clinically relevant association with the risk of all-cause and SSc-related hospitalisation or death in patients with SSc-ILD in the context of the SENSCIS® trial. It also provides evidence that FVC decline may be used as a surrogate endpoint for time to first hospitalisation, a clinically relevant parameter, though further validation is required. Our analysis further suggests that slowing FVC decline may prevent hospitalisations in patients with SSc-ILD and supports the value of serial FVC measurement in randomised controlled trials.

## Supplementary Information

**Additional file 1: Supplementary Methods S1.** Estimated model coefficients for the joint model of FVC% predicted and time to first hospitalisation or death over 52 weeks. **Supplementary Methods S2.** Estimated model coefficients for the joint model of FVC% predicted and time to first SSc-related hospitalisation or death over 52 weeks. **Supplementary Methods S3.** Estimated model coefficients for the joint model of FVC% predicted and time to first ER or hospital admission followed by ICU or death over 52 weeks. **Supplementary Methods S4.** Estimated model coefficients for the joint model of FVC% predicted and time to first hospitalisation or death over the whole trial. **Supplementary Methods S5.** Estimated model coefficients for the joint model of FVC% predicted and time to first SSc-related hospitalisation or death over the whole trial. **Supplementary Methods S6.** Estimated model coefficients for the joint model of FVC% predicted and time to first ER or hospital admission followed by ICU or death over the whole trial. **Supplementary Results S7.** Summary of SSc-related medical conditions with an incidence of > 5%. **Supplementary Results S8.** Summary of baseline comorbidities with an incidence of > 5%. **Supplementary Results S9.** Summary of patient discontinuations during trial period. **Supplementary Results S10.** Association between current value of FVC% predicted and risk of first hospitalisation endpoints over 52 weeks.

## Data Availability

To ensure independent interpretation of clinical study results, Boehringer Ingelheim grants all external authors access to relevant material, including participant-level clinical study data, as needed by them to fulfil their role and obligations as authors under the International Committee of Medical Journal Editors (ICMJE) criteria. Clinical study documents and participant clinical study data are available to be shared on request after publication of the primary manuscript in a peer-reviewed journal, and if regulatory activities are complete and other criteria met per the BI Policy on Transparency and Publication of Clinical Study Data (see Medical & Clinical Trials | Clinical Research | MyStudyWindow). Bona fide, qualified scientific and medical researchers are eligible to request access to the clinical study data with corresponding documentation describing the structure and content of the datasets. Upon approval, and governed by a Legal Agreement, data are shared in a secured data-access system for a limited period of 1 year, which may be extended upon request. Prior to providing access, clinical study documents and data will be examined, and, if necessary, redacted and de-identified, to protect the personal data of study participants and personnel, and to respect the boundaries of the informed consent of the study participants. Researchers should use the https://vivli.org/ link to request access to study data and visit Medical & Clinical Trials | Clinical Research | MyStudyWindow for further information.

## Abbreviations

ATA: Anti-topoisomerase I antibody
CI: Confidence interval
CTD: Connective tissue disease
DL_CO_: Diffusing capacity of the lung for carbon monoxide
ER: Emergency room
FVC: Forced vital capacity
HR: Hazard ratio
HRQoL: Health-related quality of life
ICU: Intensive care unit
ILD: Interstitial lung disease
IPF: Idiopathic pulmonary fibrosis
PRO: Patient-reported outcome
SD: Standard deviation
SSc: Systemic sclerosis
