# Supplementary material for: Impact of lung function decline on time to hospitalisation events in systemic sclerosis-associated interstitial lung disease (SSc-ILD): a joint model analysis
Source: Arthritis Res Ther. 2022 Jan 10;24:19. doi: 10.1186/s13075-021-02710-9 (PMC8751320; doi:10.1186/s13075-021-02710-9)
Supplement: Supplementary file 1 — Additional file 1: Supplementary Methods S1. Estimated model coefficients for the joint model of FVC% predicted and time to first hospitalisation or death over 52 weeks. Supplementary Methods S2. Estimated model coefficients for the joint model of FVC% predicted and time to first SSc-related hospitalisation or death over 52 weeks. Supplementary Methods S3. Estimated model coefficients for the joint model of FVC% predicted and time to first ER or hospital admission followed by ICU or death over 52 weeks. Supplementary Methods S4. Estimated model coefficients for the joint model of FVC% predicted and time to first hospitalisation or death over the whole trial. Supplementary Methods S5. Estimated model coefficients for the joint model of FVC% predicted and time to first SSc-related hospitalisation or death over the whole trial. Supplementary Methods S6. Estimated model coefficients for the joint model of FVC% predicted and time to first ER or hospital admission followed by ICU or death over the whole trial. Supplementary Results S7. Summary of SSc-related medical conditions with an incidence of > 5%. Supplementary Results S8. Summary of baseline comorbidities with an incidence of > 5%. Supplementary Results S9. Summary of patient discontinuations during trial period. Supplementary Results S10. Association between current value of FVC% predicted and risk of first hospitalisation endpoints over 52 weeks. [file 13075_2021_2710_MOESM1_ESM.docx]

**Supplement**

**Impact of lung function decline on time to hospitalisation events in systemic sclerosis-associated interstitial lung disease (SSc-ILD): a joint model analysis**

Michael Kreuter^1,2^*, Francesco Del Galdo^3^*, Corinna Miede^4^, Dinesh Khanna^5^, Wim A. Wuyts^6^, Laura K. Hummers^7^, Margarida Alves^8^, Nils Schoof^8^, Christian Stock^9^, Yannick Allanore^10^

^1^Center for Interstitial and Rare Lung Diseases, Pneumology and Respiratory Care Medicine, Thoraxklinik, University of Heidelberg, Heidelberg, Germany; ^2^German Center for Lung Research (DZL), Heidelberg, Germany; ^3^Scleroderma Programme NIHR BRC and Institute of Rheumatic and Musculoskeletal Medicine, University of Leeds, Leeds, United Kingdom; ^4^mainanalytics GmbH, Sulzbach/Taunus, Germany; ^5^Division of Rheumatology/Department of Internal Medicine, Scleroderma Program, University of Michigan, Ann Arbor, MI, USA; ^6^Interstitial Lung Diseases Unit, University Hospitals Leuven, Leuven, Belgium; ^7^Division of Rheumatology, Johns Hopkins University School of Medicine, Baltimore, MD, USA; ^8^Boehringer Ingelheim International GmbH, Ingelheim am Rhein, Germany; ^9^Boehringer Ingelheim Pharma GmbH & Co. KG, Ingelheim am Rhein, Germany; ^10^Department of Rheumatology A, Descartes University, APHP, Cochin Hospital, Paris, France

*Contributed equally

**Corresponding author:** Michael Kreuter, MD

**Address:** Center for Interstitial and Rare Lung Diseases, Pneumology and Respiratory Care Medicine, Thoraxklinik, University of Heidelberg, Röntgenstrasse 1, 69121 Heidelberg, Germany

**Email:** kreuter@uni-heidelberg.de

**Supplementary Methods S1.** **Estimated model coefficients for the joint model of FVC% predicted and time to first hospitalisation or death over 52 weeks**

|  | **Estimate** | **Standard error** |
| --- | --- | --- |
| Longitudinal Process: Intercept | 0.2849 | 0.6077 |
| Longitudinal Process: Slope | −2.8907 | 0.4154 |
| Longitudinal Process: TRT by slope | 1.1620 | 0.5909 |
| Longitudinal Process: ATA | 0.08791 | 0.2791 |
| Longitudinal Process: BASE | 0.9933 | 0.008157 |
| Longitudinal Process: Log SD Residual | 0.9971 | 0.01460 |
| Longitudinal Process: Log SD Random Intercept | 1.0549 | 0.03990 |
| Longitudinal Process: Log SD Random Slope | 1.7227 | 0.05229 |
| Longitudinal Process: Covariance Intercept by Slope | −0.4938 | 1.0452 |
| Event Process: Log Hazard Stratum 1 Period 1 | −7.8686 | 0.3875 |
| Event Process: Log Hazard Stratum 1 Period 2 | −8.5572 | 0.4434 |
| Event Process: Log Hazard Stratum 1 Period 3 | −8.4580 | 0.3879 |
| Event Process: Log Hazard Stratum 1 Period 4 | −8.4154 | 0.4045 |
| Event Process: Log Hazard Stratum 1 Period 5 | −8.4478 | 0.4000 |
| Event Process: Log Hazard Stratum 1 Period 6 | −8.5158 | 0.4617 |
| Event Process: Log Hazard Stratum 2 Period 1 | −8.1800 | 0.5304 |
| Event Process: Log Hazard Stratum 2 Period 2 | −7.8876 | 0.4099 |
| Event Process: Log Hazard Stratum 2 Period 3 | −8.4465 | 0.4732 |
| Event Process: Log Hazard Stratum 2 Period 4 | −8.1276 | 0.4326 |
| Event Process: Log Hazard Stratum 2 Period 5 | −7.9324 | 0.4015 |
| Event Process: Log Hazard Stratum 2 Period 6 | −7.6726 | 0.3964 |
| Association (slope-dependent) | −0.1186 | 0.02455 |

Data collected during treatment period.

*ATA*, anti-topoisomerase I antibody; *FVC*, forced vital capacity; *SD*, standard deviation; *TRT*, treatment.

**Supplementary Methods S2. Estimated model coefficients for the joint model of FVC% predicted and time to first SSc-related hospitalisation or death over 52 weeks**

|  | **Estimate** | **Standard error** |
| --- | --- | --- |
| Longitudinal Process: Intercept | 0.2614 | 0.6086 |
| Longitudinal Process: Slope | −2.7620 | 0.3986 |
| Longitudinal Process: TRT by slope | 1.4405 | 0.5641 |
| Longitudinal Process: ATA | 0.09109 | 0.2790 |
| Longitudinal Process: BASE | 0.9932 | 0.008165 |
| Longitudinal Process: Log SD Residual | 1.0094 | 0.01422 |
| Longitudinal Process: Log SD Random Intercept | 1.0536 | 0.03981 |
| Longitudinal Process: Log SD Random Slope | 1.6824 | 0.04908 |
| Longitudinal Process: Covariance Intercept by Slope | −0.1710 | 0.9780 |
| Event Process: Log Hazard Stratum 1 Period 1 | −8.4419 | 0.4992 |
| Event Process: Log Hazard Stratum 1 Period 2 | −9.7035 | 0.7364 |
| Event Process: Log Hazard Stratum 1 Period 3 | −9.2293 | 0.4573 |
| Event Process: Log Hazard Stratum 1 Period 4 | −8.8143 | 0.6129 |
| Event Process: Log Hazard Stratum 1 Period 5 | −8.9985 | 0.5329 |
| Event Process: Log Hazard Stratum 1 Period 6 | −8.8466 | 0.5280 |
| Event Process: Log Hazard Stratum 2 Period 1 | −8.9108 | 0.7423 |
| Event Process: Log Hazard Stratum 2 Period 2 | −8.3088 | 0.4948 |
| Event Process: Log Hazard Stratum 2 Period 3 | −10.5531 | 1.0216 |
| Event Process: Log Hazard Stratum 2 Period 4 | −8.0639 | 0.5391 |
| Event Process: Log Hazard Stratum 2 Period 5 | −8.7767 | 0.6017 |
| Event Process: Log Hazard Stratum 2 Period 6 | −8.6169 | 0.5994 |
| Association (slope-dependent) | −0.1299 | 0.03119 |

Data collected during treatment period.

*ATA*, anti-topoisomerase I antibody; *FVC*, forced vital capacity; *SD*, standard deviation; *SSc*, systemic sclerosis; *TRT*, treatment.

**Supplementary Methods S3. Estimated model coefficients for the joint model of FVC% predicted and time to first ER or hospital admission followed by ICU or death over 52 weeks**

|  | **Estimate** | **Standard error** |
| --- | --- | --- |
| Longitudinal Process: Intercept | 0.3348 | 0.6127 |
| Longitudinal Process: Slope | −2.9410 | 0.4093 |
| Longitudinal Process: TRT by slope | 1.3292 | 0.5865 |
| Longitudinal Process: ATA | 0.1145 | 0.2813 |
| Longitudinal Process: BASE | 0.9921 | 0.008222 |
| Longitudinal Process: Log SD Residual | 0.9944 | 0.01459 |
| Longitudinal Process: Log SD Random Intercept | 1.0539 | 0.03975 |
| Longitudinal Process: Log SD Random Slope | 1.6854 | 0.05304 |
| Longitudinal Process: Covariance Intercept by Slope | 0.9531 | 0.9881 |
| Event Process: Log Hazard Stratum 1 Period 1 | −7.6707 | 0.4034 |
| Event Process: Log Hazard Stratum 1 Period 2 | −7.1401 | 0.3615 |
| Event Process: Log Hazard Stratum 1 Period 3 | −7.9024 | 0.3579 |
| Event Process: Log Hazard Stratum 1 Period 4 | −8.3778 | 0.4246 |
| Event Process: Log Hazard Stratum 1 Period 5 | −8.2549 | 0.4225 |
| Event Process: Log Hazard Stratum 1 Period 6 | −8.4922 | 0.4223 |
| Event Process: Log Hazard Stratum 2 Period 1 | −7.3675 | 0.4275 |
| Event Process: Log Hazard Stratum 2 Period 2 | −7.7528 | 0.5904 |
| Event Process: Log Hazard Stratum 2 Period 3 | −8.2591 | 0.5140 |
| Event Process: Log Hazard Stratum 2 Period 4 | −7.9621 | 0.4252 |
| Event Process: Log Hazard Stratum 2 Period 5 | −7.6576 | 0.3956 |
| Event Process: Log Hazard Stratum 2 Period 6 | −8.0214 | 0.4224 |
| Association (slope-dependent) | −0.04765 | 0.03345 |

Data collected during treatment period.

*ATA*, anti-topoisomerase I antibody; *ER*, emergency room; *FVC*, forced vital capacity; *ICU*, intensive care unit; *SD*, standard deviation; *TRT*, treatment.

**Supplementary Methods S4. Estimated model coefficients for the joint model of FVC% predicted and time to first hospitalisation or death over the whole trial**

|  | **Estimate** | **Standard error** |
| --- | --- | --- |
| Longitudinal Process: Intercept | 0.2259 | 0.6095 |
| Longitudinal Process: Slope | −2.8932 | 0.3468 |
| Longitudinal Process: TRT by slope | 1.0732 | 0.4976 |
| Longitudinal Process: ATA | 0.1510 | 0.2793 |
| Longitudinal Process: BASE | 0.9941 | 0.008186 |
| Longitudinal Process: Log SD Residual | 1.0156 | 0.01343 |
| Longitudinal Process: Log SD Random Intercept | 1.0540 | 0.03920 |
| Longitudinal Process: Log SD Random Slope | 1.5387 | 0.05294 |
| Longitudinal Process: Covariance Intercept by Slope | 0.5377 | 0.8551 |
| Event Process: Log Hazard Stratum 1 Period 1 | −7.9276 | 0.3345 |
| Event Process: Log Hazard Stratum 1 Period 2 | −8.5586 | 0.3964 |
| Event Process: Log Hazard Stratum 1 Period 3 | −8.3645 | 0.3448 |
| Event Process: Log Hazard Stratum 1 Period 4 | −8.6882 | 0.4004 |
| Event Process: Log Hazard Stratum 1 Period 5 | −8.5741 | 0.3709 |
| Event Process: Log Hazard Stratum 1 Period 6 | −8.4265 | 0.3673 |
| Event Process: Log Hazard Stratum 2 Period 1 | −8.1534 | 0.4392 |
| Event Process: Log Hazard Stratum 2 Period 2 | −7.9409 | 0.3706 |
| Event Process: Log Hazard Stratum 2 Period 3 | −8.3247 | 0.4047 |
| Event Process: Log Hazard Stratum 2 Period 4 | −7.8199 | 0.3417 |
| Event Process: Log Hazard Stratum 2 Period 5 | −7.9936 | 0.3514 |
| Event Process: Log Hazard Stratum 2 Period 6 | −7.8775 | 0.3470 |
| Association (slope-dependent) | −0.1289 | 0.02819 |

Data collected during treatment period.

*ATA*, anti-topoisomerase I antibody; *FVC*, forced vital capacity; *SD*, standard deviation; *TRT*, treatment.

**Supplementary Methods S5. Estimated model coefficients for the joint model of FVC% predicted and time to first SSc-related hospitalisation or death over the whole trial**

|  | **Estimate** | **Standard error** |
| --- | --- | --- |
| Longitudinal Process: Intercept | 0.2023 | 0.6139 |
| Longitudinal Process: Slope | −2.7812 | 0.3300 |
| Longitudinal Process: TRT by slope | 1.1896 | 0.4710 |
| Longitudinal Process: ATA | 0.1572 | 0.2809 |
| Longitudinal Process: BASE | 0.9942 | 0.008240 |
| Longitudinal Process: Log SD Residual | 1.0316 | 0.01303 |
| Longitudinal Process: Log SD Random Intercept | 1.0622 | 0.03880 |
| Longitudinal Process: Log SD Random Slope | 1.4968 | 0.04912 |
| Longitudinal Process: Covariance Intercept by Slope | 0.5947 | 0.7978 |
| Event Process: Log Hazard Stratum 1 Period 1 | −8.5813 | 0.4336 |
| Event Process: Log Hazard Stratum 1 Period 2 | −9.8196 | 0.5457 |
| Event Process: Log Hazard Stratum 1 Period 3 | −8.9843 | 0.4929 |
| Event Process: Log Hazard Stratum 1 Period 4 | −8.6916 | 0.4508 |
| Event Process: Log Hazard Stratum 1 Period 5 | −9.0543 | 0.4431 |
| Event Process: Log Hazard Stratum 1 Period 6 | −9.6365 | 0.6006 |
| Event Process: Log Hazard Stratum 2 Period 1 | −8.7128 | 0.5497 |
| Event Process: Log Hazard Stratum 2 Period 2 | −9.3666 | 0.5477 |
| Event Process: Log Hazard Stratum 2 Period 3 | −8.7648 | 0.5428 |
| Event Process: Log Hazard Stratum 2 Period 4 | −8.6351 | 0.5336 |
| Event Process: Log Hazard Stratum 2 Period 5 | −9.2785 | 0.6000 |
| Event Process: Log Hazard Stratum 2 Period 6 | −8.4945 | 0.4335 |
| Association (slope-dependent) | −0.1563 | 0.03677 |

Data collected during treatment period.

*ATA*, anti-topoisomerase I antibody; *FVC*, forced vital capacity; *SD*, standard deviation; *SSc*, systemic sclerosis; *TRT*, treatment.

**Supplementary Methods S6. Estimated model coefficients for the joint model of FVC% predicted and time to first ER or hospital admission followed by ICU or death over the whole trial**

|  | **Estimate** | **Standard error** |
| --- | --- | --- |
| Longitudinal Process: Intercept | 0.1879 | 0.6192 |
| Longitudinal Process: Slope | −2.8997 | 0.3345 |
| Longitudinal Process: TRT by slope | 1.1770 | 0.4842 |
| Longitudinal Process: ATA | 0.1806 | 0.2834 |
| Longitudinal Process: BASE | 0.9941 | 0.008312 |
| Longitudinal Process: Log SD Residual | 1.0283 | 0.01332 |
| Longitudinal Process: Log SD Random Intercept | 1.0647 | 0.03892 |
| Longitudinal Process: Log SD Random Slope | 1.4752 | 0.05188 |
| Longitudinal Process: Covariance Intercept by Slope | 1.5623 | 0.7984 |
| Event Process: Log Hazard Stratum 1 Period 1 | −7.6475 | 0.3780 |
| Event Process: Log Hazard Stratum 1 Period 2 | −7.6752 | 0.3271 |
| Event Process: Log Hazard Stratum 1 Period 3 | −7.7387 | 0.3269 |
| Event Process: Log Hazard Stratum 1 Period 4 | −7.9868 | 0.3945 |
| Event Process: Log Hazard Stratum 1 Period 5 | −8.8769 | 0.4235 |
| Event Process: Log Hazard Stratum 1 Period 6 | −8.4277 | 0.3521 |
| Event Process: Log Hazard Stratum 2 Period 1 | −7.1837 | 0.3749 |
| Event Process: Log Hazard Stratum 2 Period 2 | −8.5590 | 0.5902 |
| Event Process: Log Hazard Stratum 2 Period 3 | −8.3069 | 0.5150 |
| Event Process: Log Hazard Stratum 2 Period 4 | −7.4379 | 0.3740 |
| Event Process: Log Hazard Stratum 2 Period 5 | −8.0094 | 0.3526 |
| Event Process: Log Hazard Stratum 2 Period 6 | −8.4274 | 0.4196 |
| Association (slope-dependent) | −0.03879 | 0.04042 |

Data collected during treatment period.

*ATA*, anti-topoisomerase I antibody; *ER*, emergency room; *FVC*, forced vital capacity; *ICU*, intensive care unit; *SD*, standard deviation; *TRT*, treatment.

**Supplementary Results S7. Summary of SSc-related medical conditions with an incidence of > 5%**

|  | **Patients included in joint models**  **(*N* = 574)** | **Patients with  all-cause hospitalisation events or death (*n* = 78)** | **Patients with  SSc-related hospitalisation events or death (*n* = 42)** | **Patients with admission to ER or hospital followed by admission to ICU or death**  **(*n* = 75)** |
| --- | --- | --- | --- | --- |
| Raynaud phenomenon |  |  |  |  |
| Yes − In the past | 555 (96.7) | 72 (92.3) | 38 (90.5) | 71 (94.7) |
| Yes − Still at screening | 506 (88.2) | 66 (84.6) | 34 (81.0) | 65 (86.7) |
| Digital ulcers |  |  |  |  |
| Yes − In the past | 222 (38.7) | 32 (41.0) | 19 (45.2) | 26 (34.7) |
| Yes − Still at screening | 67 (11.7) | 10 (12.8) | 7 (16.7) | 7 (9.3) |
| Synovitis |  |  |  |  |
| Yes − In the past | 138 (24.0) | 13 (16.7) | 6 (14.3) | 24 (32.0) |
| Yes − Still at screening | 59 (10.3) | 3 (3.8) | 1 (2.4) | 11 (14.7) |
| Joint contractures |  |  |  |  |
| Yes − In the past | 143 (24.9) | 17 (21.8) | 8 (19.0) | 18 (24.0) |
| Yes − Still at screening | 125 (21.8) | 15 (19.2) | 6 (14.3) | 16 (21.3) |
| Friction rubs |  |  |  |  |
| Yes − In the past | 54 (9.4) | 4 (5.1) | 0 (0.0) | 8 (10.7) |
| Yes − Still at screening | 39 (6.8) | 4 (5.1) | 0 (0.0) | 6 (8.0) |
| CK elevation |  |  |  |  |
| Yes − In the past | 60 (10.5) | 14 (17.9) | 11 (26.2) | 6 (8.0) |
| Yes − Still at screening | 22 (3.8) | 8 (10.3) | 6 (14.3) | 1 (1.3) |
| Weakness (muscles) |  |  |  |  |
| Yes − In the past | 111 (19.3) | 13 (16.7) | 7 (16.7) | 16 (21.3) |
| Yes − Still at screening | 76 (13.2) | 7 (9.0) | 3 (7.1) | 11 (14.7) |
| Muscle atrophy |  |  |  |  |
| Yes − In the past | 38 (6.6) | 8 (10.3) | 4 (9.5) | 7 (9.3) |
| Yes − Still at screening | 34 (5.9) | 7 (9.0) | 4 (9.5) | 6 (8.0) |
| Oesophageal (dysphagia, reflux) |  |  |  |  |
| Yes − In the past | 426 (74.2) | 59 (75.6) | 27 (64.3) | 63 (84.0) |
| Yes − Still at screening | 359 (62.5) | 50 (64.1) | 24 (57.1) | 57 (76.0) |
| Stomach (early satiety, vomiting) |  |  |  |  |
| Yes − In the past | 127 (22.1) | 18 (23.1) | 9 (21.4) | 20 (26.7) |
| Yes − Still at screening | 87 (15.2) | 13 (16.7) | 7 (16.7) | 14 (18.7) |
| Diarrhoea (malabsorption, bact. overgrowth) |  |  |  |  |
| Yes − In the past | 103 (17.9) | 11 (14.1) | 6 (14.3) | 17 (22.7) |
| Yes − Still at screening | 59 (10.3) | 4 (5.1) | 3 (7.1) | 8 (10.7) |
| Bloating |  |  |  |  |
| Yes − In the past | 98 (17.1) | 11 (14.1) | 5 (11.9) | 16 (21.3) |
| Yes − Still at screening | 69 (12.0) | 7 (9.0) | 3 (7.1) | 12 (16.0) |
| Constipation |  |  |  |  |
| Yes − In the past | 114 (19.9) | 15 (19.2) | 8 (19.0) | 16 (21.3) |
| Yes − Still at screening | 69 (12.0) | 8 (10.3) | 4 (9.5) | 8 (10.7) |
| Incontinence |  |  |  |  |
| Yes − In the past | 38 (6.6) | 8 (10.3) | 5 (11.9) | 8 (10.7) |
| Yes − Still at screening | 27 (4.7) | 6 (7.7) | 4 (9.5) | 5 (6.7) |
| Hypertension |  |  |  |  |
| Yes − In the past | 152 (26.5) | 26 (33.3) | 18 (42.9) | 25 (33.3) |
| Yes − Still at screening | 124 (21.6) | 21 (26.9) | 13 (31.0) | 22 (29.3) |
| Proteinuria (+ or more) |  |  |  |  |
| Yes − In the past | 21 (3.7) | 4 (5.1) | 3 (7.1) | 3 (4.0) |
| Yes − Still at screening | 8 (1.4) | 1 (1.3) | 1 (2.4) | 0 (0.0) |
| Palpitations |  |  |  |  |
| Yes − In the past | 95 (16.6) | 16 (20.5) | 9 (21.4) | 24 (32.0) |
| Yes − Still at screening | 45 (7.8) | 7 (9.0) | 5 (11.9) | 12 (16.0) |
| Conduction blocks |  |  |  |  |
| Yes − In the past | 33 (5.7) | 4 (5.1) | 1 (2.4) | 8 (10.7) |
| Yes − Still at screening | 30 (5.2) | 2 (2.6) | 1 (2.4) | 7 (9.3) |
| Diastolic function abnormal |  |  |  |  |
| Yes − In the past | 42 (7.3) | 5 (6.4) | 3 (7.1) | 4 (5.3) |
| Yes − Still at screening | 40 (7.0) | 5 (6.4) | 3 (7.1) | 4 (5.3) |
| Pulmonary hypertension |  |  |  |  |
| Yes − In the past | 52 (9.1) | 7 (9.0) | 6 (14.3) | 9 (12.0) |
| Yes − Still at screening | 42 (7.3) | 5 (6.4) | 4 (9.5) | 8 (10.7) |

Data are *n* (%) unless otherwise stated.

*CK*, creatine kinase; *ER*, emergency room; *ICU*, intensive care unit; *SSc*, systemic sclerosis.

**Supplementary Results S8. Summary of baseline comorbidities with an incidence of > 5%**

|  | **Patients included in joint models**  **(*N* = 574)** | **Patients with  all-cause hospitalisation events or death (*n* = 78)** | **Patients with  SSc-related hospitalisation events or death (*n* = 42)** | **Patients with admission to ER or hospital followed by admission to ICU or death**  **(*n* = 75)** |
| --- | --- | --- | --- | --- |
| Infections and infestations | 113 (19.7) | 19 (24.4) | 10 (23.8) | 16 (21.3) |
| Neoplasms benign, malignant and unspecified (including cysts and polyps) | 66 (11.5) | 6 (7.7) | 4 (9.5) | 7 (9.3) |
| Blood and lymphatic system disorders | 56 (9.8) | 11 (14.1) | 6 (14.3) | 8 (10.7) |
| Anaemia | 20 (3.5) | 4 (5.1) | 2 (4.8) | 2 (2.7) |
| Immune system disorders | 92 (16.0) | 11 (14.1) | 8 (19.0) | 11 (14.7) |
| Seasonal allergy | 43 (7.5) | 6 (7.7) | 5 (11.9) | 6 (8.0) |
| Endocrine disorders | 99 (17.2) | 16 (20.5) | 10 (23.8) | 13 (17.3) |
| Hypothyroidism | 61 (10.6) | 8 (10.3) | 7 (16.7) | 8 (10.7) |
| Metabolism and nutrition disorders | 191 (33.3) | 24 (30.8) | 11 (26.2) | 23 (30.7) |
| Hyperlipidaemia | 42 (7.3) | 5 (6.4) | 3 (7.1) | 4 (5.3) |
| Hypercholesterolaemia | 40 (7.0) | 5 (6.4) | 2 (4.8) | 3 (4.0) |
| Vitamin D deficiency | 40 (7.0) | 3 (3.8) | 2 (4.8) | 6 (8.0) |
| Dyslipidaemia | 31 (5.4) | 2 (2.6) | 0 | 6 (8.0) |
| Obesity | 11 (1.9) | 5 (6.4) | 3 (7.1) | 4 (5.3) |
| Psychiatric disorders | 131 (22.8) | 16 (20.5) | 9 (21.4) | 17 (22.7) |
| Insomnia | 53 (9.2) | 5 (6.4) | 2 (4.8) | 9 (12.0) |
| Depression | 49 (8.5) | 5 (6.4) | 4 (9.5) | 2 (2.7) |
| Anxiety | 30 (5.2) | 5 (6.4) | 2 (4.8) | 5 (6.7) |
| Sleep disorder | 6 (1.0) | 3 (3.8) | 3 (7.1) | 1 (1.3) |
| Nervous system disorders | 119 (20.7) | 19 (24.4) | 12 (28.6) | 23 (30.7) |
| Carpal tunnel syndrome | 20 (3.5) | 5 (6.4) | 2 (4.8) | 6 (8.0) |
| Neuropathy peripheral | 7 (1.2) | 4 (5.1) | 2 (4.8) | 4 (5.3) |
| Headache | 28 (4.9) | 2 (2.6) | 2 (4.8) | 7 (9.3) |
| Eye disorders | 79 (13.8) | 10 (12.8) | 6 (14.3) | 9 (12.0) |
| Dry eye | 29 (5.1) | 4 (5.1) | 4 (9.5) | 4 (5.3) |
| Cardiac disorders | 102 (17.8) | 22 (28.2) | 13 (31.0) | 16 (21.3) |
| Mitral valve incompetence | 11 (1.9) | 5 (6.4) | 2 (4.8) | 3 (4.0) |
| Tricuspid valve incompetence | 10 (1.7) | 4 (5.1) | 1 (2.4) | 2 (2.7) |
| Vascular disorders | 222 (38.7) | 32 (41.0) | 15 (35.7) | 37 (49.3) |
| Varicose vein | 15 (2.6) | 1 (1.3) | 0 | 4 (5.3) |
| Respiratory, thoracic and mediastinal disorders | 233 (40.6) | 35 (44.9) | 17 (40.5) | 37 (49.3) |
| Sleep apnoea syndrome | 22 (3.8) | 5 (6.4) | 2 (4.8) | 4 (5.3) |
| Rhinitis allergic | 28 (4.9) | 1 (1.3) | 0 | 4 (5.3) |
| Gastrointestinal disorders | 301 (52.4) | 42 (53.8) | 20 (47.6) | 42 (56.0) |
| Hiatus hernia | 29 (5.1) | 4 (5.1) | 2 (4.8) | 2 (2.7) |
| Hepatobiliary disorders | 35 (6.1) | 6 (7.7) | 3 (7.1) | 4 (5.3) |
| Skin and subcutaneous tissue disorders | 121 (21.1) | 19 (24.4) | 9 (21.4) | 22 (29.3) |
| Musculoskeletal and connective tissue disorders | 293 (51.0) | 41 (52.6) | 18 (42.9) | 40 (53.3) |
| Arthralgia | 76 (13.2) | 7 (9.0) | 3 (7.1) | 11 (14.7) |
| Osteoarthritis | 44 (7.7) | 7 (9.0) | 1 (2.4) | 8 (10.7) |
| Osteoporosis | 42 (7.3) | 6 (7.7) | 3 (7.1) | 4 (5.3) |
| Back pain | 21 (3.7) | 5 (6.4) | 1 (2.4) | 3 (4.0) |
| Arthritis | 13 (2.3) | 0 | 0 | 4 (5.3) |
| Renal and urinary disorders | 52 (9.1) | 5 (6.4) | 2 (4.8) | 9 (12.0) |
| Nephrolithiasis | 11 (1.9) | 1 (1.3) | 0 | 4 (5.3) |
| Renal cyst | 8 (1.4) | 1 (1.3) | 1 (2.4) | 4 (5.3) |
| Reproductive system and breast disorders | 76 (13.2) | 7 (9.0) | 4 (9.5) | 16 (21.3) |
| Erectile dysfunction | 19 (3.3) | 4 (5.1) | 1 (2.4) | 5 (6.7) |
| Benign prostatic hyperplasia | 14 (2.4) | 1 (1.3) | 1 (2.4) | 4 (5.3) |
| Congenital, familial and genetic disorders | 27 (4.7) | 6 (7.7) | 2 (4.8) | 7 (9.3) |
| General disorders and administration site conditions | 73 (12.7) | 10 (12.8) | 5 (11.9) | 15 (20.0) |
| Pain | 12 (2.1) | 6 (7.7) | 4 (9.5) | 3 (4.0) |
| Oedema peripheral | 14 (2.4) | 2 (2.6) | 1 (2.4) | 5 (6.7) |
| Investigations | 85 (14.8) | 11 (14.1) | 2 (4.8) | 9 (12.0) |
| Echocardiogram | 35 (6.1) | 2 (2.6) | 0 | 1 (1.3) |
| Injury, poisoning and procedural complications | 37 (6.4) | 6 (7.7) | 2 (4.8) | 7 (9.3) |
| Surgical and medical procedures | 143 (24.9) | 22 (28.2) | 11 (26.2) | 23 (30.7) |
| Hysterectomy | 44 (7.7) | 11 (14.1) | 7 (16.7) | 10 (13.3) |
| Appendicectomy | 12 (2.1) | 4 (5.1) | 2 (4.8) | 3 (4.0) |
| Social circumstances | 203 (35.4) | 16 (20.5) | 12 (28.6) | 21 (28.0) |
| Post-menopause | 137 (23.9) | 12 (15.4) | 9 (21.4) | 15 (20.0) |
| Menopause | 62 (10.8) | 2 (2.6) | 2 (4.8) | 6 (8.0) |

Data are *n* (%) unless otherwise stated. Conditions described in the SSc-related history have been excluded.

*ER*, emergency room; *ICU*, intensive care unit; *SSc*, systemic sclerosis.

**Supplementary Results S9. Summary of patient discontinuations during trial period**

|  | **Patients included in joint models**  **(*N* = 574)** | **Patients with  all-cause hospitalisation events or death (*n* = 78)** | **Patients with  SSc-related hospitalisation events or death (*n* = 42)** | **Patients with admission to ER or hospital followed by admission to ICU or death**  **(*n* = 75)** |
| --- | --- | --- | --- | --- |
| Prematurely discontinued from trial medication before 52 weeks | 85 (14.8) | 19 (24.4) | 12 (28.6) | 14 (18.7) |
| Adverse events | 59 (10.3) | 15 (19.2) | 11 (26.2) | 9 (12.0) |
| Worsening of disease under study | 11 (1.9) | 4 (5.1) | 4 (9.5) | 0 (0.0) |
| Worsening of other pre-existing disease | 2 (0.3) | 1 (1.3) | 1 (2.4) | 1 (1.3) |
| Other adverse events^a^ | 46 (8.0) | 10 (12.8) | 6 (14.3) | 8 (10.7) |
| Non-compliant with protocol | 2 (0.3) | 0 (0.0) | 0 (0.0) | 1 (1.3) |
| Lost to follow-up | 0 (0.0) | 0 (0.0) | 0 (0.0) | 0 (0.0) |
| Patient refusal to continue taking  trial medication | 16 (2.8) | 3 (3.8) | 1 (2.4) | 4 (5.3) |
| Other | 8 (1.4) | 1 (1.3) | 0 (0.0) | 0 (0.0) |

Data are *n* (%) unless otherwise stated.

^a^ Including adverse events related to study medication.

*ER*, emergency room; *ICU*, intensive care unit; *SSc*, systemic sclerosis.

**Supplementary Results S10. Association between current value of FVC% predicted and risk of first hospitalisation endpoints over 52 weeks**

|  | **Time to first all-cause hospitalisation or death**  **(*n* = 568)** | **Time to first SSc-related hospitalisation or death**  **(*n* = 570)** | **Time to first admission to ER or admission to hospital followed by admission to ICU or death**  **(*n* = 572)** |
| --- | --- | --- | --- |
| **Longitudinal sub-model^a^** | | | |
| Estimated slope difference nintedanib  vs. placebo (95% CI) | 1.29 (0.14, 2.43) | 1.38 (0.28, 2.48) | 1.38 (0.24, 2.53) |
| *P* value | 0.03 | 0.01 | 0.02 |
| **Time to event sub-model^b^** | | | |
| Number of patients with event, *n* (%) | 78 (13.7) | 42 (7.4) | 75 (13.1) |
| **Difference in FVC% predicted, HR (95% CI)** | | | |
| 1-unit decrease | 1.01 (0.99, 1.02) | 1.02 (1.00, 1.03) | 1.01 (1.00, 1.02) |
| 3-unit decrease | 1.02 (0.98, 1.06) | 1.05 (0.99, 1.11) | 1.03 (0.99, 1.07) |
| 5-unit decrease | 1.04 (0.97, 1.11) | 1.08 (0.98, 1.18) | 1.04 (0.97, 1.12) |
| P-value | 0.29 | 0.13 | 0.22 |

Data collected during treatment period using current value parameterisation.

^a^ Random effects normal linear model of FVC% predicted with predictor variables ATA status and FVC% predicted at baseline, a separate slope for patients on treatment, trajectories modelled by a linear trend, and an unstructured variance−covariance matrix.

^b^ Piecewise exponential baseline hazard, stratified by ATA status, and endogenous time-dependent covariate FVC% predicted as estimated current value of the longitudinal response.

*ATA*, anti-topoisomerase antibody; *CI*, confidence interval; *ER*, emergency room; *FVC*, forced vital capacity; *HR*, hazard ratio, *ICU*, intensive care unit; *SSc*, systemic sclerosis.
